# Type 1 Monteggia Equivalent Fracture with Ipsilateral Distal Radius Fracture: A Case Report

**DOI:** 10.5704/MOJ.2503.016

**Published:** 2025-03

**Authors:** YS Gokceoglu, EY Ozger

**Affiliations:** 1 Department of Orthopaedics and Traumatology, Mehmet Akif Inan Education and Research Hospital, Sanliurfa, Turkey; 2 Department of Physiotherapy and Rehabilitation, Istanbul University-Cerrahpasa, Istanbul, Turkey

**Keywords:** Monteggia fracture dislocation, paediatric forearm injuries, ipsilateral distal radius fracture, Monteggia equivalent

## Abstract

In this case report, we present a 13-years-old patient who sustained a Monteggia equivalent fracture along with an ipsilateral distal radius fracture following a fall on the elbow. Comminuted ulnar fracture was treated with open reduction and internal fixation with a bridging plate. After restoring the ulnar length, the radial neck fracture was successfully reduced. The distal radius fracture was managed conservatively. Our literature review shows that, the patient is one of the comparatively older patients treated with open reduction and internal fixation in this area and that a successful outcome was achieved with early mobilisation. This case underscores the need for further studies to determine the optimal treatment strategy in such cases.

## Introduction

Monteggia equivalent fractures are a range of forearm injuries that differ from the classic Monteggia fracture-dislocation pattern. These injuries can present in various ways, necessitating a thorough understanding for an accurate diagnosis and successful treatment. Type 1 Monteggia equivalent fractures involve proximal third ulnar fractures and radial neck fractures rather than radial head dislocation^1^.

Ipsilateral distal radius and type 1 Monteggia equivalent fractures are rare and complex injuries that require a comprehensive treatment approach. These fractures are uncommon in clinical practice, and only a few cases are documented in the literature. A thorough treatment plan is necessary to stabilise the proximal forearm and address the distal injury, ensuring full restoration of arm function^2^.

This report aims to present our strategy for treating a 13-year-old patient who suffered from a type 1 Monteggia equivalent fracture and an ipsilateral distal radius fracture. We will discuss the diagnostic difficulties, potential treatment options, and reasoning behind our management approach and review the existing literature on similar cases. In doing so, we hope to emphasise the significance of identifying this unusual injury combination to improve patient outcomes.

## Case Report

A 13-year-old boy was brought to the emergency room after falling on the left elbow at school, resulting in swelling of the wrist and elbow. Examination revealed palpable distal pulses with no neurological deficits. Radiographs revealed a distal radius fracture, comminuted proximal ulnar fracture, and radial neck fracture. The patient was diagnosed with a type 1 Monteggia equivalent injury and ipsilateral distal radius fracture. The patient was immobilised with an above-elbow splint and admitted to the orthopaedic department for further management and surgical planning.

The surgical procedure commenced with the patient lying in the supine position under general anaesthesia. An incision measuring 7cm was made on the posterolateral aspect of the patient's arm. The fracture site was then accessed, and the ulnar and proximal radius fractures were reduced through the application of traction, valgus, and supination manoeuvres. The fracture was then fixed in place using a 10-hole tubular plate with screws placed in two proximal and three distal positions. After the surgery was completed, intra-operative fluoroscopy images showed that the radial head was successfully reduced and that there was no displacement of the distal radius fracture. The surgical wound was then closed, and the patient's elbow was immobilised at a 90° angle with the forearm in a neutral position.

**Fig. 1:**
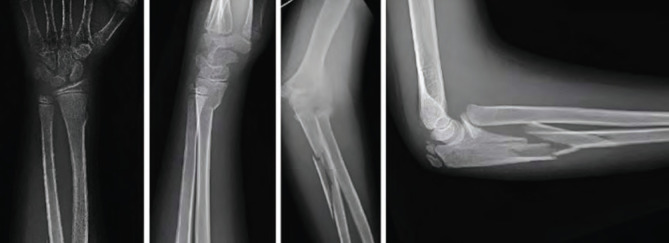
Pre-operative anteroposterior and lateral radiographs showing a Monteggia equivalent fracture characterised by a comminuted fracture of the proximal ulnar diaphysis and a radial neck fracture accompanied by an ipsilateral distal radius fracture.

**Fig. 2:**
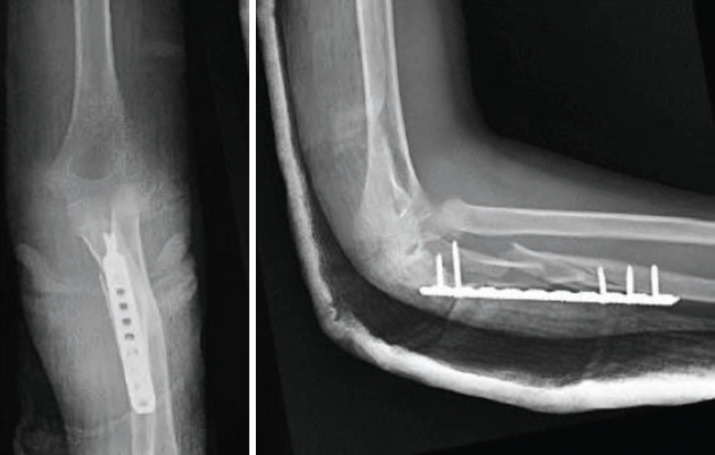
Post-operative radiographs at two weeks demonstrating satisfactory alignment of the ulnar shaft with internal fixation using bridging plate and screws. The radial neck fracture was adequately reduced, and there was no displacement of the distal radial fracture.

**Fig. 3:**
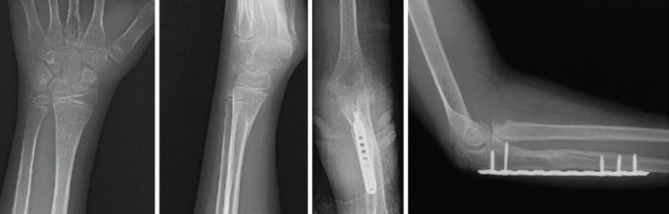
Six-month post-operative radiographs showing complete healing of the ulnar and radial fractures with maintained alignment and hardware in situ. Full restoration of the anatomical relationship between the ulna, radial head, and distal radius was noted.

After 17 days, the stitches and splints were taken off, and physical therapy began. At the 1-month mark, the patient showed natural hand movements and a range of elbow movements from 0° to 120°, including full pronation and supination. At the 7-week mark, the patient demonstrated elbow range of motion from 0° to 140°, with equal bilateral pronation and supination. At the 6-month mark, the patient showed elbow range of motion from 0° to 160°, with equal bilateral pronation and supination. The follow-up graphs are presented in (Fig. 1, 2 and 3).

## Discussion

Monteggia and Monteggia equivalent fractures result from complex mechanisms that can arise from both direct and dynamic forces^3^. One theory suggests that a direct impact on the forearm leads to ulnar fractures and radial head dislocations due to deformation. Alternatively, excessive rotation of the radius over the ulna can cause radial head dislocation or proximal radius fractures, along with ulnar shaft fractures. These mechanisms can also result from a combination of hyperextension and biceps pull, leading to radial head dislocation and ulnar fractures due to tension from body weight. Effective treatment and management of these injuries require a thorough understanding of their multifaceted nature^4^.

The instance of a Type III Monteggia fracture dislocation and an ipsilateral epiphyseal fracture of the distal radius in a child, as described in the case report by Kapil Mani *et al*^2^, is an exceedingly rare and intricate injury. This type of injury can render the diagnosis more difficult and increase the likelihood of overlooking crucial aspects during the initial assessment. In this particular case, a ten-year-old boy was misdiagnosed in the emergency room but received appropriate treatment with closed reduction and K-wire fixation. This case underscores the importance of meticulous examination and radiographic evaluation in the management of acute forearm injuries in children, particularly when dealing with swelling and pain that can obscure other injuries. Successful treatment of the Monteggia lesion through surgical intervention resulted in full bony union and restored wrist and elbow motion within three months.

Our research underscores the importance of a thorough initial assessment of paediatric forearm injuries to identify all components. We employed open reduction and internal fixation with a plate for the ulna, while Kapil Mani *et al* used closed reduction and K-wire fixation in their case^2^. This difference in treatment approaches highlights the diverse surgical options available for managing complex forearm injuries in children. It emphasises the need to tailor the technique to the specific injury pattern and overall condition of the patient. Both cases highlighted the potential for missed injuries in complex fractures and the importance of comprehensive diagnostic protocols and flexible treatment strategies to ensure optimal outcomes. The ongoing debate over best management practices for such injuries underscores the need for further research and discussion within the orthopaedic community^5^.

In a study on rare forearm fractures, the treatment involved the use of titanium elastic nails and Kirschner wires, whereas our approach used open reduction and internal fixation with a plate and screws^1^. This highlights the ongoing debate regarding the most effective strategies for complex forearm fractures in paediatric patients. Our case demonstrates the benefits of securing the ulnar length and its role in aligning associated radial fractures. However, the successful use of less invasive stabilisation techniques in this study also showed benefits in certain fracture patterns. Further research is needed to establish more definitive guidelines for treating Monteggia equivalent fractures and associated forearm injuries given the variability in injury presentations and outcomes.
